# Examining Electronic Medical Record Migration in a Team-Based Primary Care Clinic: Case Study

**DOI:** 10.2196/87759

**Published:** 2026-08-10

**Authors:** Max Moloney, Madura Sundareswaran

**Affiliations:** 1 Centre for Studies in Primary Care, Queen's University, Kingston, ON, Canada; 2 School of Medicine, Faculty of Education and Health Sciences, University of Limerick, Limerick, Munster, V94 T9PX, Ireland, 353 061-234-850; 3 Department of Family Medicine, Peterborough-Kawartha Site, Queen's University, Peterborough, ON, Canada

**Keywords:** electronic medical records, primary care, case study, quality improvement, physician leadership, change management

## Abstract

**Background:**

Electronic medical record (EMR) systems are now ubiquitous in Ontario primary care, with near-universal adoption among family physicians. EMRs have become critical pieces of clinic infrastructure that can affect nearly every aspect of clinical and administrative work. As EMR vendors consolidate and functionality evolves, some clinics undertake EMR migrations—complex transitions that require data conversion, workflow redesign, training, and change management. Despite their importance and wide adoption, there is a gap in understanding how community-based primary care organizations in Canadian settings navigate migration in practice, particularly from the perspective of frontline clinicians and staff.

**Objective:**

The project aimed to describe a single case of EMR migration within an 11-physician, team-based Family Health Organization (FHO) in Peterborough, Ontario, from the perspective of frontline clinicians and staff. This included assessing the factors influencing the decision to migrate, the operational and human factors that shaped implementation, and practical lessons for supporting and evaluating future EMR transitions.

**Methods:**

A single-site, physician-led quality improvement project was conducted in collaboration with the Peterborough Ontario Health Team using an exploratory case study approach. This approach was used to examine a complex, real-world health IT transition in its local organizational context. This project examined one FHO’s migration between EMR providers using purposive sampling of individuals directly involved in the transition, including a lead physician (n=1), a lead administrator (n=1), family physicians (n=9), interprofessional health care providers (n=12), and medical office assistants (n=9). Data sources included 2 semistructured interviews, 2 focus groups, and 1 comprehensive document review. Data collection spanned the planning, implementation, and stabilization phases of the migration from February 2022 to November 2024. Sessions were recorded, transcribed, and deidentified. Documents included emails, meeting minutes, training communications, project planning materials, and troubleshooting records. Interview and focus group transcripts were analyzed thematically. Study documents were reviewed separately to construct a narrative chronology of the migration.

**Results:**

Four interrelated themes emerged: (1) burden of invisible labor, (2) cost and uncertainty as a catalyst, (3) importance of physician leadership, and (4) engage stakeholders early. Participants described migration as a resource-intensive organizational transition shaped by unpaid preparation work, cost pressures, clinical governance, quality assurance, and the responsiveness of internal and external stakeholders, particularly the vendor.

**Conclusions:**

This study illuminates the process of an EMR migration within one team-based FHO. Effective transitions depend on resourcing the often-invisible work of change, sustaining physician-led governance, and securing early, collaborative engagement with vendors and external partners. These findings complement provincial migration guidance by emphasizing protected time for training and quality assurance, local workflow adaptation, staged validation to shorten the transition period (“gray zone”), and explicit assessment of vendor responsiveness. Lessons are readily actionable for similar group practices and clinics planning future migrations or optimizations.

## Introduction

In Canada, the term electronic medical record (EMR) refers to a computerized patient health record system often used within a physician’s practice to support clinical care delivery and practice operations [1]. Rates of EMR adoption continue to rise across Canada, and in 2024, Canada Health Infoway found that 97% of family physicians used an EMR [2]. In Ontario, family physicians and group practices select and implement their own EMR systems, supported by OntarioMD, a subsidiary of the Ontario Medical Association [3]. OntarioMD certifies EMR offerings that meet provincial requirements for use in primary care, including technical and interoperability requirements. Despite the availability of more than a dozen certified EMR vendors in Ontario, the following 3 vendors dominated the market: Telus Health Solutions Inc, QHR Technologies Inc, and WELL EMR Group Inc. In 2025, these 3 vendors alone accounted for 84% of the market share [4]. In Ontario, individual physicians and practices are responsible for selecting, implementing, and, if required, migrating between EMR systems for their clinical use.

Early EMR adoption and implementation have been well documented in both the United States and Canada over the last 20 years [5-7], with most literature focusing on the transition from paper-based charts to EMRs. However, since initial EMR adoption, group practices and clinics across Ontario have completed EMR migrations—the process of moving a health care organization’s EMR from one system to another. The earliest and largest instance of this in Ontario was documented by Fisher [8] in 2013, in which a team of 46 physicians migrated from one EMR provider to a different one. Although EMR migrations are known to occur in Ontario primary care settings, they are infrequently described despite their relevance to the needs of clinicians today. EMR migrations are recognized as complex, high-risk endeavors requiring significant resources [9]. Beyond the expected tasks, such as extracting, mapping, and validating data, practices must also redesign workflows, train staff, and manage staff during periods of transition [9]. In a 2023 systematic review of transitions from one EMR to another, Miake-Lye et al [10] identified 40 studies, of which only 11 addressed provider perspectives; only 2 included information on satisfaction and lessons learned; and 3 discussed satisfaction and perceptions of the new EMR. Many of the studies came from large institutions with limited generalizability to smaller outpatient clinics, and only 1 study was Canadian [10]. This review recommended considering human factors and mixed methods approaches to capture the organizational change in EMR transitions.

The outcomes of EMR transitions are largely shaped by the experiences of frontline workers [11]. In the Canadian context, a 2025 study by Taneja et al [12] found that user experiences of EMR implementation varied based on local configuration decisions and on the extent to which the system aligned with existing workflows. Similarly, Zharima et al [11] found that successful EMR implementation depended on effective engagement strategies that help stakeholders understand, participate in, and sustain the work of change. However, this critical frontline perspective is underrepresented in the existing literature.

Case studies are increasingly recognized as a qualitative research methodology and lend themselves well to explaining specific phenomena, with a focus on human behavior, opinions, and attitudes, and to understanding the effects of an event on people [13]. Successful implementation of health IT cannot be focused solely on technical solutions and should include organizational issues, project management, and human behavior [14].

When one Family Health Organization (FHO) of 11 family physicians in Peterborough, Ontario, shared its decision to migrate to a new EMR in 2024, it presented an opportunity to study and understand the migration process in real time. Although migrations occur routinely across the province, this project adopted an exploratory case study approach to describe an EMR migration from the perspective of frontline workers within a single FHO from the time the decision was made to switch EMR vendors to 1 month after deployment. The goal of this project is to describe a single case of EMR migration, explore the factors that influenced the decision to migrate, and highlight lessons learned from EMR migration in a small, community-based family practice setting. The project also aims to provide a framework for evaluating EMR migrations to support health care organizations in evaluating and reviewing such complex processes internally.

## Methods

### Overview

This project used an exploratory case study approach to provide an in-depth description of an EMR migration in a single-site, real-life context. In collaboration with the Peterborough Ontario Health Team (POHT), this approach aimed to describe the experience of one EMR migration in Ontario, Canada, starting from the decision to switch EMRs through to 1 month after deployment. The project was designed as a local quality improvement (QI) initiative with the goal of maximizing success in EMR migration processes in similar practice settings. This approach used routinely collected operational data, involved no randomization or experimental intervention, posed minimal risk, and was conducted for local service improvement rather than hypothesis-testing or generalizable research.

Despite growing support for case study methodology to study IT implementation in health care settings, no single framework exists for this work, thereby creating variability in the quality and reproducibility of available data to date [13]. However, consistent recommendations for case study research include acquiring multiple sources of data, triangulation, iterative analysis, and consideration of multisite or collective case studies [13].

We collected multiple sources of data in alignment with the case study framework proposed by Yin [15]. Semistructured interviews and focus groups were conducted to better understand the factors influencing the decision to migrate and to illustrate the potential complexity of the migration process. Document review was conducted separately to construct a chronological account of the migration process and to describe the sequence of administrative, planning, training, implementation, and stabilization activities. Reporting was guided by the COREQ (Consolidated Criteria for Reporting Qualitative Research) checklist, which is provided as Checklist 1.

### Context

A decision to pursue an EMR migration was made by a group of 11 family physicians, who were part of a multidisciplinary FHO in Peterborough, Ontario. The migration process was led by a designated FHO physician lead and lead administrator (nonphysician). At the time of migration, the clinical team was composed of 11 physicians, 9 medical office assistants (MOAs), 1 lead administrator, and 12 interprofessional health care providers (IHPs) including nurses, nurse practitioners, pharmacists, and mental health clinicians. This FHO served a total of approximately 13,000 patients.

The core research team consisted of 1 family physician (primary investigator), who was a participant in this EMR migration, and 1 research assistant. Employees of the POHT with qualitative research experience were also involved in project development and design. Including a physician investigator who was familiar with the migration process and real-world clinical implications allowed for in-depth reflexive methodology, including the development of the research question and questionnaires, and the analysis of the data. This also supported a more inclusive and tailored approach to participant recruitment. The engagement and establishment of a research team provided the opportunity for rich discussions supporting collaborative reflexivity [16].

### Focus Groups and Interviews

Case study methodology should include multiple sources of evidence to provide a comprehensive, in-depth picture of the numerous elements of a particular case. In the absence of a pre-existing framework to evaluate IT systems implementation in health care settings, an open-ended, semistructured interview guide was developed collaboratively by the primary investigator (a family physician) and the staff members of the POHT. Questionnaire development focused on the proposed research question, built on existing literature, and considered early discussions during the development of the interview guide (Multimedia Appendix 1). The questions were designed to be open-ended and flexible, allowing participants to guide the discussions, as appropriate. The use of interviews allowed more in-depth recounting of the events leading up to and during the EMR migration, with more context than would be available from document review alone [17]. One-on-one interviews were offered to the core leadership team (1 lead physician and 1 lead administrator) to allow for discussion of more sensitive or confidential matters, to guide additional questions for the focus groups, and to provide detailed information regarding timelines [15,17].

Beyond the core leadership team of the FHO, as discussed above, the multidisciplinary clinical team consisted of 10 additional family physicians and 21 administrative and clinical nonphysician staff. To support a thorough and rich opportunity for data collection, the research team conducted facilitated focus groups. Two distinct groups were proposed to ensure that there were no supervisory relationships (ie, physician and nonphysician groups) and to target an ideal focus group size, as defined by Leung and Savithiri [18], of 7 to 10 participants. The interview guides for both one-on-one interviews and focus groups were intended as a framework, recognizing that additional data gathered from this process may strengthen future questionnaire development.

The interviews were conducted by an employee of the POHT who was not affiliated with the FHO being studied to minimize bias. All interviews aimed to obtain information relevant to premigration, migration, and postmigration, with a focus on preparation, workload, challenges, and lessons learned. The interview guides are all available in Multimedia Appendix 1.

### Recruitment and Sampling

A purposive sampling strategy was used to recruit frontline staff with direct involvement in EMR use and in the migration process for focus groups. Potential participants were identified through the FHO administrator, and all eligible participants were invited by email. Two focus groups were scheduled. The team’s lead physician and FHO administrator were invited via email to participate in individual, semistructured interviews intended to provide in-depth details regarding the migration timeline and to understand the preparation work needed for this process. Prior to participation, a consent letter was shared with all participants, and consent was implied upon active participation. Compensation was provided to participants in the form of an honorarium if work was completed outside of regular working hours. Final participants included the lead physician (n=1), the lead administrator (n=1), family physicians (n=9), IHPs (n=12), and MOAs (n=9), with one family physician (n=1) not participating in the study due to their role on the research team. The final interviews conducted included a lead physician interview (n=1), a lead administrator interview (n=1), a physician group interview (n=9), and an IHP interview (n=12).

### Document Review

One frequently cited method of data collection in case study methodology is document review, but it has primarily been used to triangulate with additional sources of data. In case study methodology, documents may include meeting minutes, email correspondence, internal memos, planning materials, and workflow diagrams [15]. For the purposes of this study, document review was solely used to provide a narrative, chronological account of events pertaining to the EMR migration. The FHO lead and FHO administrator were asked to share any documents they believed would objectively illustrate the administrative tasks and timelines involved in planning and implementing the EMR migration. Suggestions for documents included email correspondence, meeting minutes, internal documents and spreadsheets, and correspondence regarding training. A total of 545 documents were received, including internal clinic meeting minutes, vendor correspondence, training communications, project planning emails, and troubleshooting discussions.

### Data Analysis

A total of 2 focus groups and 2 semistructured interviews were completed. Each session lasted 45 to 60 minutes; was conducted via Microsoft Teams; and was audio-recorded, transcribed, and deidentified. All recordings were transcribed verbatim by an employee of the POHT and validated by a research assistant from the research team. Identifying details were removed during transcription, and each participant was assigned a random, nonidentifiable number.

The analysis of data from interviews and focus groups followed the 6-phase framework for thematic analysis by Braun and Clarke [19], and all analyses were completed by the core research team. The researchers independently reviewed the anonymized transcripts to familiarize themselves with the data, and they were asked to track notes and comments in the comments section of the transcripts. Each analyst was encouraged to document any reflective observations and highlight any quotations that illustrated frontline workers’ unique experiences of the EMR migration, aligning with an inductive approach. Data were independently coded to identify recurring patterns in relation to the research question. Each researcher produced a list of codes, which were then compared. The researchers met to discuss any discrepancies, and consensus was reached on code definitions. A final codebook was created after rereview, discussion, and ongoing familiarization with the data. The final codebook is available in Multimedia Appendix 2.

Coding was completed in Microsoft Word, and assigned codes were compared line by line to ensure consistency and to prompt discussion to ensure alignment in the understanding of each code. Relevant excerpts from all transcripts were extracted into a single document presenting a compilation of quotes categorized by code. Similar codes were grouped together, and each researcher independently identified themes and then shared their findings for discussion. The process of identifying, revising, and searching for themes was iterative, with ongoing revisions and discussions by both researchers. Relevant excerpts from transcripts were extracted and categorized by theme, which highlighted redundancies and allowed for further refinement. A final list of themes was created and agreed upon by 2 members of the research team once saturation was met. These themes were then compared against the dataset to ensure they were reflective and comprehensive.

Data from the documents received were used to construct a narrative chronology of the migration to corroborate and extract specific details from the discussions and timelines shared during the leadership one-on-one interviews. Data from the interviews and focus groups provided broad timelines and highlighted key components of the migration process, but to gain a deeper understanding of the administrative and planning work required, supplementary document analysis was conducted. The extraction of time stamps and dates provided more accurate timelines than interviews and focus groups alone. Key elements of the migration were identified in the interviews, and the documents were searched retrospectively to pull quotes and dates after familiarization with their content. Thematic analysis was not conducted on the documents; instead, these data were used to conduct an observational case study within the project, “storying” the case, as described by Greenhalgh et al [20], and to summarize a chronological account of important actions and events.

### Ethical Considerations

This project was reviewed by the Queen’s University Health Sciences and Affiliated Teaching Hospitals Research Ethics Board, and a formal exemption was granted from research ethics board review (TRAQ 6044565). It was granted an exemption under Tri-Council Policy Statement Article 2.5: “Quality assurance and quality improvement studies, program evaluation activities, and performance reviews, or testing within normal educational requirements when used exclusively for assessment, management or improvement purposes, do not constitute research for the purposes of this policy, and do not fall within the scope of REB review.” All transcripts were deidentified prior to analysis, and all relevant documents were stored in a password-protected OneDrive (Microsoft Corp) accessible only to team members to protect confidentiality.

## Results

### Chronology: Understanding the Process

**Figure 1.**
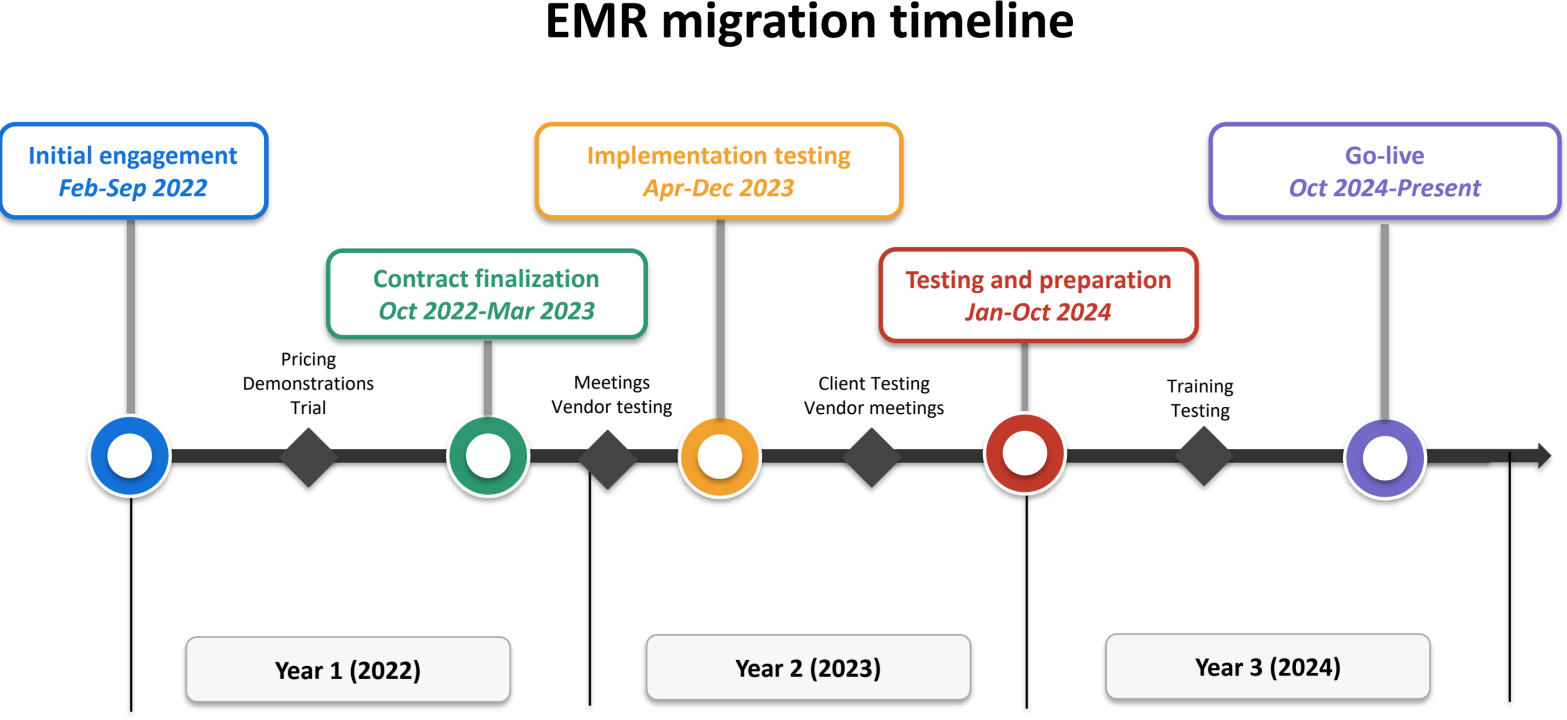
Electronic medical record (EMR) migration timeline.

Document review was used to construct a chronological account of the EMR migration (Figure 1). Documents were first screened for relevance to the EMR migration and organized chronologically by date. Key dates, decisions, implementation activities, training events, vendor communications, data transfer milestones, and stabilization activities were then compiled into a timeline. Documents were not used to generate codes or themes. The migration emerged as a prolonged and resource-intensive process shaped not only by implementation activities but also by the circumstances that led the clinic to pursue the transition.

Interest in an EMR migration was first expressed by this group of family physicians in February 2022 after meeting minutes highlighted concerns about the current system. Drivers for the migration included rising and unpredictable costs, lack of response from the current vendor, loss of support for the existing server, and uncertainty about the future and possible EMR consolidation. These factors were not merely background context—they shaped the urgency and planning of the migration itself and were repeatedly referenced by participants when explaining why the transition unfolded as it did. Concerns raised about the EMR migration included potentially accelerating physician retirements or delaying opportunities for physician recruitment. Records indicated that this transition involved a subgroup of physicians attending early product demonstrations from 5 other EMR vendors and engaging in discussions with other clinics to gather feedback on their existing EMRs.

In April 2022, after multiple demonstrations, a subgroup of physicians shared their experiences with the entire physician team, and a group-wide demonstration of the preferred EMR was organized. In June 2022, 2 additional physicians were given access to a “sandbox environment” of the potential new EMR. In September 2022, 6 months after the initial expression of interest, documents revealed more in-depth discussion and evaluation of the potential new EMR. Strengths of the favored product included functionality, ease of use, good workflow, built-in features such as faxing and a patient messaging portal at no extra cost, inclusion of licensing for support staff, and the prospect of better support from the new vendor. Concerns and risks related to this migration included a lack of familiarity and integration in Ontario, an issue that was unique to this particular EMR. The FHO committed to moving forward with the migration to the new vendor in October 2022.

A distinguishing feature of this migration was that the selected EMR was not yet OntarioMD certified when it was first considered. This was explicitly considered during the decision-making process in April 2022 as a limitation, but it was not viewed as a major deterrent because interim workarounds were available, including faxed hospital records and access to Connecting Ontario. Certification delays continued to shape the migration timeline. By September 2023, the OntarioMD certification was expected to take an additional 9 to 12 months. During implementation, reports and electronic laboratory results were managed through workarounds, including fax-based routing, Connecting Ontario, and auditing of incoming reports. By the stabilization period, documents described ongoing monitoring with the vendor actively supporting troubleshooting, allowing the clinic to continue the migration while integration and certification were addressed concurrently.

From a review of the communications, it was evident that this EMR migration was notably more challenging, given the vendor’s limited experience in Ontario. However, this was understood from the beginning as an introductory email from the vendor acknowledged that this migration was “unique” and described the migration process as a “collaboration.” Multiple meetings were proposed over the next several months to discuss a deployment plan, training, and priorities for product development. These records described the migration as highly collaborative and reliant on frequent communication between the staff and the vendor.

The documents outlined numerous factors that influenced the timeline of this EMR migration. These were identified as: time for data extraction, loss of updates for the existing server, further product development to better meet the needs of Ontario family physicians, testing of data transfers, decluttering of the current EMR (eg, removing redundant and duplicate documents), uploading and sharing province and community-specific forms to the new EMR, and quality assurance testing. The chronology of the documents showed that much of the migration work occurred outside of routine patient care and required substantial invisible labor related to quality assurance of the data.

An internal communication was sent to all staff in the FHO in August 2024 with confirmation of a “go-live” date (September 2024), details regarding in-person training, and a suggestion to consider booking appointments more lightly for 9 days from that date. In September 2024, a communication was distributed to all staff members outlining the training process. A series of training videos, created specifically for clinicians and clerical staff, along with practice exercises, was provided by the vendor. A 3-hour in-person training day was also organized for all physicians, administrative staff, and IHPs.

A final time and date were selected after which point no new data could be input into the old EMR, and access to the old EMR was considered “read-only.” All staff were informed of a process known as the “gray zone” wherein use of the new EMR must begin, but not all data would be completely transferred—therefore, historical data would be missing in patient charts during use, and referencing the old EMR would be necessary. The vendor also shared with all users the order in which the data migration would happen, outlining top priorities, including patient demographics, appointments, and documents, with lower priorities being billing data. The staged transition process was frequently highlighted as one of the most challenging phases of migration in focus group discussions.

On-site support by the vendor to assist with the transition was offered from September 30 to October 10. During this time, multiple emails were sent at the end of the day with updates, tips for use, and additional resources and tools. Daily meetings were held, which the vendor described as an opportunity to “discuss what is going well and our challenges. Please raise the things you find abnormal as early as possible in the project [so] that we can fix it for you and your colleagues.” These communications described daily troubleshooting and demonstrated that the vendor was responsive during the transition.

A final email served as a wrap-up prior to the deployment team leaving the site. Additional on-site training was made available again 4 to 6 weeks after the migration. The migration was completed in November 2024.

### Thematic Analysis

Four interrelated end-user themes were identified from interview and focus group transcripts: (1) burden of invisible labor, (2) cost and cost uncertainty as a catalyst, (3) importance of physician leadership, and (4) engage stakeholders early (Table 1).

**Table 1.** Key themes.

Theme	Description
Burden of invisible labor	Switching systems forced users to unlearn habits, rebuild “muscle memory,” and remap searches. Much of the preparation, work, and communication took place after hours.
Cost and uncertainty as a catalyst	Rising and opaque vendor costs strained physician-run clinics and prompted the group to evaluate alternatives and proceed with migration.
Importance of physician leadership	Physician leads coordinated vendors, governance, and safety-sequencing decisions, quality assurance, and data mapping, while also managing risks.
Engage stakeholders early	Early involvement of clinicians, staff, laboratories, and a responsive vendor smoothed training, reduced support needs, and preserved continuity during cutover.

#### Theme 1: Burden of Invisible Labor

Switching between different systems required users to unlearn entrenched habits, rebuild “muscle memory,” and remap search behaviors before speed and efficiency returned. Clinicians repeatedly framed the first weeks as a deliberate slowing of clinical work while they relearned basic functions and workflows, describing it as a cognitively demanding process:

*It’s almost like you’re almost learning to drive again...things that I could do in 20 seconds [were] taking me 3 minutes...times that by 30 patients...you’re just going to be slower and that just takes time.*

[Focus Group 1]

Searching for important data, which would have previously been easy or effortless, required much more effort and time. Often, routine work spilled outside the visit:

*And it’s a fast-paced job that we do...I was staying an extra hour and a half at the end of the night just to make sure you stay on top of for the next day. Lot of extra time.*

[Focus Group 2]

Many participants discussed the difficulty in accurately quantifying the number of hours required to prepare and conduct the EMR migration. Much of the work was informal and not documented systematically: chart reorganization, template curation, quality assurance, and regular communication to all members of the team often occurred after hours and between patients. One participant summarized, “A lot of evening meetings...replying to emails...checking out workflows and communicating them to the team...” (Interview 1).

For most outpatient family practices, such an endeavor is often considered a cost of doing business. An EMR migration is a significant investment of time and resources and an undertaking that is uncompensated and unrecognized for most family physicians. As 1 participant noted, “The success of this project relies on physicians being willing to do unpaid work...we all went to training that was unpaid” (Interview 1).

#### Theme 2: Cost and Uncertainty as a Catalyst

Direct financial expenses and vendor terms, particularly with the outgoing EMR vendor, were difficult to pin down prospectively and shaped the strategy. Physicians described the financial stress of running small businesses, emphasizing that the cost of basic clinic necessities, including an EMR, is directly borne by the physicians. As such, rising costs and uncertainty strongly influenced the decision to consider and move forward with an EMR migration, as noted by 1 participant:

*The EMR costs—when [previous vendor] advised us that they were moving everybody from a solo server to an ASP platform—went up 100%...it’s not sustainable. They started charging for administrative use...a license for nurses...everything that [new vendor] has within the EMR is included in the price....*

[Interview 2]

Physicians also described indirect financial effects from reduced efficiency, staff overtime, and potentially lower billings during the transition. One user stated, “Most of our [earnings] are going to be down by the sheer volume decrease that we’re seeing with this process” (Focus Group 1).

Physicians repeatedly flagged the baseline cost increase, opacity, and shifting licensing fees as determinants of their decision to migrate. This uncertainty reinforced the push to explore alternatives, as described by 1 participant:

*We actually spent probably several months before even looking at new EMRs trying to get a straight answer from [vendor] about how much their platform was going to cost, when we were going to be forced to make the switch, and details around what that would look like. It was very, very difficult to get.*

[Interview 1]

#### Theme 3: Importance of Physician Leadership

Physician leaders provided governance, translating clinical realities into sequencing and safety decisions while coordinating vendors and internal teams.

*It was [lead physician’s] responsibility to find all of the options...get demos, create comparison spreadsheets and figure out the top ones...engage with the vendor, get contract prices...[and] make sure that we understood everything that they were offering.*

[Interview 1]

Quality assurance was explicitly resourced, with physicians allocating substantial time to this task. The role was described as operational and communicative:

*The lead role is really one of putting out fires and kind of communicating...we were meeting every Thursday...as we got closer, those meetings increased as needed.*

[Interview 1]

Participants also credited the scale and quality of the leadership team for the migration. Moving legacy information into discrete, clinically reliable fields was technically and operationally challenging, with direct safety implications, participants noted:

*We started doing data exports on Fridays...identifying it, and trying to figure out where to map it in [new EMR] because the functionalities are very different.*

[Interview 2]

The result was an extended gray zone, with both EMRs activated, which lasted much longer because of the complexity of the data conversion, during which teams relied on manual verification and interim workarounds. Physician leadership also owned risk identification and mitigation during the migration:

*[You want] to be sure that there’s somebody who fully understands the medicine of it at the table...there’s always a risk of losing data. I’m always more worried about inaccurate data than missing data.*

[Interview 1]

This highlighted the importance of physician leadership during the EMR migration, given their unique perspective on understanding the risks present during the transition.

#### Theme 4: Engage Stakeholders Early

Users who were engaged early in decision-making, such as through demos and workflow mapping, described smoother experiences and needed less support than staff who were engaged later in the transition process:

*I think we should have more training, like for a couple of weeks before, even the month before, to start. So we could start even just playing with it on our own....*

[Focus Group 2]

Continuity through the transition depended on active communication with hospitals, laboratory partners, and a collaborative vendor.

*[Labs] actually have to work with the EMR provider...[our] project manager worked directly with the contact at Excelleris for the actual cutover...and the same with Dynacare...they monitored and watched on their end to make sure our labs were being pushed through to the EMR.*

[Interview 2]

Some staff felt that broader early involvement may have improved both practical readiness and emotional buy-in before implementation:

*If you had a committee of people that were able to look at different options and give feedback and make a decision...employees would have felt valued and like that have a bit of a say.*

[Focus Group 2]

This underscores the value of early contact and an understanding of timelines between organizations. Additionally, a strong vendor partnership assisted with problem-solving, “[The new vendor] were very, very responsive. I couldn’t have asked for a better company to work with” (Interview 2).

This was repeatedly cited as a decisive factor in the overall migration experience. Early and specific engagement between frontline users and vendors reduced friction and supported effective implementation during migration.

## Discussion

### Principal Findings

This study describes a single case of EMR migration in a community-based Ontario FHO, examining the factors that influenced the decision to migrate, the lessons learned during implementation, and a practical framework for health care organizations seeking to internally evaluate complex EMR migration processes. During the exploration of the EMR migration in real time, 4 interrelated themes emerged. The findings from this study suggest that EMR migration in community primary care is not simply a technical exercise in replacing software; rather, it changes the entire workflow process and requires physician leadership, operational flexibility, and sustained coordination among multiple actors.

Three key takeaways follow and are consistent with the previous literature. First, physician leadership and engagement across all stages are key determinants of a safe and effective EMR migration. Clinical leaders understand the inherent risks of an EMR migration, work directly with programs, and routinely liaise with various stakeholders. This aligns with OntarioMD’s migration guidance [3] and the practice recommendation of Fisher [8] on designated leads and structured change management. In this study, physician leadership extended beyond governance alone and included vendor communication, quality assurance, and mitigation of patient safety risks during the gray zone period. This is important, as community clinics may have limited time and support for physician leadership of a migration; however, their importance in ensuring a successful migration is critical.

Second, training and data conversion are crucial for patient safety and are not merely clerical tasks. Hands-on and specific training near activation, appropriate planning and scheduling, and staged validation shortened the gray zone and reduced the time required to complete the EMR migration. These elements are emphasized in OntarioMD’s transition guide [3]. These findings reinforce that role-specific training and dedicated training time are central to safe implementation. Participants described the gray zone as one of the most difficult periods of the transition, and interview data showed that manual verification and adaptations were required to maintain continuity of care.

Vendor engagement materially shapes workload, cost, and safety. The transition literature highlights the role of functionality and organizational change as drivers of EMR switches, while other literature highlights an innovation vacuum that can shift the burden to local teams [21]. This case demonstrates how a responsive vendor and a positive relationship can offset these dynamics. In both Canada and the United States, a small number of vendors hold substantial market share, raising concerns about pricing and responsiveness [2]. The broader transition literature highlights the importance of organizational change in shaping the user experience during an EMR transition [10,12]. In this case, a responsive vendor relationship appeared to reduce friction during migration by supporting daily troubleshooting and hosting regular meetings to resolve any issues with the EMR. This finding is consistent with more recent implementation literature suggesting that local workflow integration and collaborative configuration are important factors in the success of an EMR migration [12].

This study also demonstrates the value of examining EMR migration as a complex process dependent on both technical and social factors. Crowe et al [22] describe case-study methodology as useful in health services research for generating an in-depth understanding of complex issues in real-world settings, especially when asking how and why an intervention is implemented and experienced. Similarly, Gill and Borycki [13] argue that EMR implementation case studies are valuable because their success depends not only on technical functionality but also on the organizational context, project management, and human behavior. This study reconstructed the decision to migrate and the experience of those who participated in the migration. Although the findings are specific to this unique context, they offer practical lessons and provide a framework for other small health care organizations considering an EMR migration.

The finding that the motivations for migration remained central throughout the transition also warrants emphasis. In this case, concerns about cost, vendor responsiveness, and technological sustainability shaped not only the decision to migrate but also the degree of urgency and tolerance for disruption during implementation. This suggests that future studies and migration planning frameworks should consider pretransition pressures as part of the migration process itself, rather than treating them solely as background context.

### Future Directions

As a single-site QI study, generalizability to all EMR migrations was not a goal of this study; however, the mechanisms for an effective EMR migration are actionable for similar Ontario practices and provide a framework for future evaluations in community settings. Future research may consider applying this framework across multiple sites and including a broader range of data sources, such as direct observation notes and performance measures before and after the migration. Specific attention may be given to quantitative metrics such as the number of appointments per day before, during, and after the migration. Future studies may adapt the interview and focus group questions depending on practice contexts and explicitly triangulate document review with interview findings. Reflexivity should be encouraged among all clinician researchers, especially if they are actively involved in the EMR migration process. Consideration may be given to standardizing questions, journaling, or reporting methods to ensure consistency in how reflexivity is captured in this type of research.

### Limitations

This was a single-site, physician-led QI project in one Ontario FHO. Because no established framework existed to evaluate this EMR migration, the research team applied a case study approach to describe this unique phenomenon. Although reflexivity was encouraged, the inclusion of a physician in the research team risked introducing bias but also provided important contextual insights that strengthened the practical evaluation of the migration. Findings relied primarily on qualitative self-reports and document review, which were subject to recall and social desirability bias. Cost figures and time estimates were specific to the clinic and were not independently validated. Additionally, the vendor context and local interoperability constraints may have limited applicability in other settings. Despite these limitations, the use of multiple data sources allowed data comparison and provided a richer understanding of how the migration unfolded.

### Conclusions

This study shows that EMR migration in community-based primary care is a complex organizational transition shaped by hidden implementation labor, health system pressures, physician leadership, and early stakeholder engagement. In this case, understanding why the clinic chose to migrate was essential to understanding how the transition unfolded, including the degree of urgency, the planning burden, and the governance structures used to manage risk. For similar practices, the most actionable implications are to recognize and resource the invisible work of migration, provide role-specific training close to go-live, involve clinical leadership directly in decision-making and quality assurance, and engage internal and external stakeholders early.

## Supplementary material

10.2196/87759 Multimedia Appendix 1 Interview guides.

10.2196/87759 Multimedia Appendix 2 Codebook.

10.2196/87759 Checklist 1 COREQ checklist.

## Abbreviations

COREQ: Consolidated Criteria for Reporting Qualitative Research
EMR: electronic medical record
FHO: Family Health Organization
IHP: interprofessional health care provider
MOA: medical office assistant
POHT: Peterborough Ontario Health Team
QI: quality improvement
