# Supplementary material for: Examining Electronic Medical Record Migration in a Team-Based Primary Care Clinic: Case Study
Source: JMIR Form Res. 2026 Aug 10;10:e87759. doi: 10.2196/87759 (PMC13458545; doi:10.2196/87759)
Supplement: Supplementary file 1 [file formative-v10-e87759-s001.pdf]

EMR Migration Focus Group Interview Guide with Administrative Staff (MOAs/RPNs/RNs/Clerical Staff) and IHPs (pharmacists/social workers/nurse practitioners).

Introduction

Thank you for agreeing to participate in a discussion about the recent EMR migration that took place in your practice. The purpose of this interview is to gain a deeper understanding of your experience during the transition to the new system, including what went well, any challenges you encountered, and the support you received. Please note that our focus is on the migration process itself; while there may have been unique challenges associated with implementing a new EMR in Ontario, this project aims to understand the general experience of switching from one EMR to another and its impact on patient care and clinicians.

Your insights will help us document the migration process and identify key areas for improvement. This conversation should take about 30-45 minutes and will be audio-recorded. Participation is completely voluntary, and you may withdraw at any time without any consequences. Your responses will remain anonymous, and no personally identifiable information will be included in the final report.

[*OBTAIN VERBAL CONSENT TO CONTINUE]*

Are you ok to proceed with the interview?

Do you have any questions before we start?

[NOTE THAT RECORDING WILL START NOW]

General Questions

1. How would you describe overall experience with the migration process?

System Usability and Patient Management

2. How did the new EMR impact your workflow and day-to-day tasks?
    - *Prompt only:* Were there any major changes to your workflow
3. What impacts, if any, did the EMR migration have on your interactions with patients?
4. What impact, if any, did switching EMRs have on your patients directly?
5. What risks did you foresee going into the EMR migration and what steps did you take, if any, to try to mitigate some of this.

Pain Points

6. Were there any unexpected challenges or particularly frustrating issues during the migration?
    a) Prompt only: Did any parts of the system or process stand out as being particularly cumbersome or confusing?

Support and Training

7. Did you receive training or support prior to using the new system?
    - What aspects of training did you feel were important for the success of the migration?
    - Were there parts of the training that you did not think were important?

Improvements and Recommendations

8. If you could change one thing about the migration process, what would it be?
9. As frontline staff in a primary care facility, what elements do you think are most important for an EMR in primary care?
10. **FOR IHP Only:** *How can the Peterborough Family Health Team or other organizations that you may be part of support its employees during an EMR migration in a FHO?*

Conclusion

11. Is there anything else you would like to share about your experience that we haven't covered?

Thank you once again for your time and valuable insights. Your feedback will be instrumental in helping to refine the process of EMR transitions.

[STOP RECORDING]

EMR Migration Structured Interview Guide for Physicians

Introduction

Thank you for agreeing to participate in a discussion about the recent EMR migration that took place in your practice. The purpose of this interview is to gain a deeper understanding of your experience during the transition to the new system, including what went well, any challenges you encountered, and the support you received. Please note that our focus is on the migration process itself; while there may have been unique challenges associated with implementing a new EMR in Ontario, this project aims to understand the general experience of switching from one EMR to another and its impact on patient care and clinicians.

Your insights will help us document the migration process and identify key areas for improvement. This conversation should take about 30-45 minutes and will be audio-recorded. Participation is completely voluntary, and you may withdraw at any time without any consequences. Your responses will remain anonymous, and no personally identifiable information will be included in the final report.

[*OBTAIN VERBAL CONSENT TO CONTINUE]*

Are you ok to proceed with the interview?

Do you have any questions before we start?

[NOTE THAT RECORDING WILL START NOW]

General Questions

1. How would you describe your overall experience with the migration process?
2. How was your day-to-day workflow impacted during the migration?
3. What impacts, if any, did the EMR migration have on your interactions with patients?
4. What risks did you foresee going into the EMR migration and what steps did you take, if any, to try to mitigate some of this.
5. What impact, if any, did your EMR migration have on other local healthcare or community organizations?
6. Did you feel supported by other organizations, advocacy groups or associations in your EMR migration? How could such organizations support EMR migrations for independent primary care providers in the future?

Training and Education:

7. What aspects of training or support did you feel were important for the success of the migration? Were there parts of the training or support that you did not think were important?
    - Prompt: Was onsite training helpful?

Clinical Workflow Integration

8. What are the biggest challenges in learning to use a new EMR?
9. Has the EMR migration had any direct or indirect financial implications for you as a physician?
10. Has the EMR migration impacted the number of hours you work per week? By how much? For how long?

Improvements and Recommendations

11. As frontline staff in a primary care facility, what elements do you think are most important for an EMR in primary care?

Conclusion

12. Is there anything else you would like to share about your experience that we haven't covered?

Thank you once again for your time and valuable insights. Your feedback will be instrumental in helping to refine the process of EMR transitions and the experiences of physicians.

[STOP RECORDING]

EMR Migration Structured Interview Guide: 1:1 with FHO Lead & FHO Administrator

Introduction

Thank you for agreeing to participate in a discussion about the recent EMR migration that took place in your practice. The purpose of this interview is to gain a deeper understanding of your experience during the transition to the new system, including what went well, any challenges you encountered, and the support you received. Please note that our focus is on the migration process itself; while there may have been unique challenges associated with implementing a new EMR in Ontario, this project aims to understand the general experience of switching from one EMR to another and its impact on patient care and clinicians.

Your insights will help us document the migration process and identify key areas for improvement. This conversation should take about 45-60 minutes and will be audio-recorded. Participation is completely voluntary, and you may withdraw at any time without any consequences. Your responses will remain anonymous, and no personally identifiable information will be included in the final report.

[*OBTAIN VERBAL CONSENT TO CONTINUE]*

Are you ok to proceed with the interview?

Do you have any questions before we start?

[NOTE THAT RECORDING WILL START NOW]

Leadership Role and Responsibilities:

1. Can you describe the scope of your responsibilities as the lead physician/administrator as they related to the EMR migration?

2. How did you ensure that staff and other members of the practice were prepared for the migration?

3. How did you work with the vendor throughout the migration process?

4. How did the migration impact your workload as a lead or administrator for the FHO?

5. Has the EMR migration impacted the number of hours you work (as administrator or lead) per week? By how much? For how long?

EMR Selection:

1. What factors were considered as part of the decision-making process for transitioning to a different EMR?

2. How does the new EMR integrate with other software and digital health tools your team uses, and how did this impact the decision to transition? If integration was not possible, what workarounds did you implement?

3. Were there any challenges related to leaving the previous EMR vendor?

4. What costs were associated with the implementation, aside from the regular recurring costs of the system? For example, did you need to upgrade or replace hardware (e.g., printers) to be compatible with the new EMR, or were there one-time costs for integrating other digital health tools?

Migration Preparation:

1. Can you provide a general timeline of the transition including when migration preparations began?

2. What were the most challenging elements of the pre-migration work?

3. What risks did you foresee going into the EMR migration and what steps did you take, if any, to try to mitigate these risks?

EMR Transition:

1. What were the biggest challenges during the first week of transition?

2. What key factors or specific steps were most helpful in ensuring success after the transition?

3. Have staff been formally scheduled for additional training since go-live? If yes, how many sessions and for what length of time?

4. What impact, if any, did your EMR migration have on other local healthcare or community organizations?

5. Did you feel supported by other organizations, advocacy groups or associations in your EMR migration? How could such organizations support EMR migrations for independent primary care providers in the future?

Reflections and Recommendations:

1. In your opinion, what went well during the migration process?

2. Is there anything that could have been done differently to make the migration smoother?

3. How do you feel about the clinic's current level of adaptation to the new EMR?

Conclusion

1. Is there anything else you would like to share about your experience that we haven't covered?
