# Supplementary material for: Examining Electronic Medical Record Migration in a Team-Based Primary Care Clinic: Case Study
Source: JMIR Form Res. 2026 Aug 10;10:e87759. doi: 10.2196/87759 (PMC13458545; doi:10.2196/87759)
Supplement: Supplementary file 2 [file formative-v10-e87759-s002.pdf]

Code	Subcodes (if applicable)
Time Investment	
EMR Familiarization	
Data Migration	Searching
	Errors
Transition Planning	Training
	Preparation
	Prioritization
Financial Cost	
Staff Impacts	Uncertainty
	Reactions
Engagement & Communication	Vendors
	Community Partners
	Staff
Patient Care	
Progress	
Lack of Progress	
Risk Tolerance & Mitigation	

Definition
Time invested to complete EMR migration
Requirement to familiarize with new EMR in practice
Examples of continuously needing to search or look for things
Presence of errors and implications in workflow
Re-training to use a new system
Work required in advance of the EMR migration
Need to prioritize the EMR migration within the clinic
Financial cost of completing the EMR migration
Feeling of uncertainty or juggling concerns around 'not knowing'
Emotional reactions or responses to the pending change, transition, etc.
Engagement with EMR vendor (old and new)
Communication with community partners such as external stakeholders
Communication with staff regarding EMR transition
Impacts of the change on patient care
Examples of changes or adaptations that are positive for the team/end user
Examples of changes or adaptations that are negative for the team/end user
Risk mitigation and management during the EMR migraiton

Code	Subcodes (if applicable)	Definition
Time Investment		Time invested to complete EMR migration

Examples
comparing their imported data to what we had, identifying problems, and making suggestions. It was a huge, huge undertaking.
We have all of our data on a local server, so there was no real technological reason why we couldn’t just take the data ourselves. So that’s what we did, and there was a lot of my time invested in basically quality
So, I invested probably a day a week for two months in doing quality assurance on Zoom with them, comparing their imported data to what we had, identifying problems, and making suggestions. It was a
Then the week that MYLE went live, we had daily meetings at the end of the day for about an hour each day for the first two weeks that I participated in, and then almost constant text exchange, where people were
We were meeting, like, probably for about six months. We were meeting once a month, and then as we got to a couple of months before, we started meeting once a week. I think we were meeting every Thursday afternoon for most of the afternoon, and then as we got closer, those meetings increased as needed. Yeah,
Yeah, more than anyone probably realizes, even in our group. I imagine I’ve invested well over 100
n. A lot of evening meetings, a lot of replying to emails, a lot of checking out workflows and communicating them to the team. Yeah, it’s sort of very hard to quantify because it’s like, at lunch, jump on this meeting or
But I don’t think anyone hired extra staff per se, but certainly, our staff maybe worked more hours, or we
So I just think recognizing the investment of unpaid time that doctors have to put in to make it work is really important, especially if you’re thinking of ever imposing it on anyone else.
Well, you're slower because you're just looking for stuff. Like before, it's almost like, it's like you're almost
Yeah, I think we were able to probably maintain like a 75% volume during the progression.
But you know, we’re in that phase where you're redoing all the individual items, and that's just a lot of extra
You don't have 5 minutes to do that, so, you know, it's just a matter of time dedicated to do, you know, EMR
So. It doesn't really affect my efficiency, but, you know, she was supposed to start work yesterday at 1, and she came in at 8 in the morning to try to catch up, right? And I don't know how long that's going to go on for.
And it's a fast-paced job that we do. Like, you’re really multitasking. And you can't seem to do that with MYLE. So, I was staying an extra hour and a half at the end of the night just to make sure you stay on top
And then there’s just—to update a file and to correct everything every visit. It's gonna take a lot longer,
I’ll say it’s the system. Because PS was so user-friendly, yes, and I don't think it's just because we've been on it for a while. For me, it's not. But I just feel like it’s a very time-consuming—
So, we had weekly meetings for months on that, and then we would do reviews, and they would come back to us and say, you know, “We need to figure out where to put this,” or they would give us an option: “Where would you like to see this?” Like messages, tasks, future things, that type of thing. So, so many months of
It was busy. I don’t think I sat down for two weeks. It was just go, go, go.
Much longer, yes. Yeah. Because we would have scrum meetings at the end of every day with the MYLE staff, the project manager, and the trainers to identify areas that were difficult for staff, where there were
So, yeah, those were for me the biggest challenges. And yes, much longer days. Lots of meetings. We would find things that weren’t right in the EMR. Then there would be meetings on how to address that.
the past six months have been the most work-heavy because that’s when we decided we’re doing it.

Code	Subcodes (if applicable)	Definition
	EMR Familiarization	Requirement to familiarize with new EMR in practice

Examples
before that. I should go back and look in my calendar—maybe even June. It was a while. It was a good couple of months at least.
Efficiency wise, probably down like 10% compared to where I was with PS, now compared to before. But I
mostly around data transfer points, even like things not coming across the way they would expect to be
you're just going to be slower and that just takes time.
I found that sending consults is something that I used to do during the appointment, that I usually save and do after for a similar reason. To have to try to compile a bunch of different documents that are hard to find.
It's like detraining your brain under one system and retraining it another way. Like, your brain always wants
And by that, I mean like learning all your shortcuts and learning how the EMR works and learning, you know, all the different things the EMR can do, because you get so caught up in clinic work,<br><br>somebody's built them to increase your billing efficiency. And then you switch to a new EMR, and you have to start again. And in that period until which you've established it—
Things such as messages, tasks, and preventative care—those were really big items that we had to figure
I had to export our address book, and then they mapped it into MYLE. So, there were just a lot of—we call them little things—that we just take for granted. You just click a button, do this, whatever, in Practice

Code	Subcodes (if applicable)	Definition
Data Migration	Searching	Examples of continuously needing to search or look for things

	Errors	Presence of errors and implications in workflow

Examples
I find like the searching for things difficult and I find having to like really learn like learning how to search for things in it. Because before, PS Suite was just so easily - you could search in any way and it would come up<br><br>I find the searching is different now, and it's all the consults, like everything that was labeled and I knew what word to search. Now I have to re-learn my vocabulary, and some of that didn't get converted at all. So, I have to individually go through other reports because I don't know what it is to find what I need<br><br>You can't easily go back into, like, a patient's chart. Like, if you're in scanning, you're doing the scanning. I have to have two MYLEs open to go back and forth. Like, prescription renewals—if the pharmacy doesn't get it in 15 minutes, they send you another one, so you get duplicates. And you want to make sure you're not sending the doctor, who is already getting so many things, 10 of the same prescription renewal. But you can't just go, "OK, go back to the patient to try," and—you have to get out, go back to the calendar, search the patient, and so in order to do that, you now have to—I have two MYLEs open on two screens, right?<br><br>I just found that they didn't know what they were missing until we called it. So, like, I'd be going through old clinician notes and then I'd let them know. And I'd be like, "OK, so just so you know, this patient was seen by this person, this person, and this person. This person's notes are in here, but these two are nowhere to be seen." And they'd be like, "Oh wait. What? What's that name?" And then they write that

data, actually. So we were really kind of attuned to that. We're realizing too, like other groups in our community, right? There's a doctor who used to be on our team who went to the Medical Center and went to Accuro. He didn't get any of his custom forms in that transition—they just didn't come, and there's no sort of way to get them.

I'm not a programmer, so it's hard to say. But yeah, I think that they have had some challenges that they weren't expecting. The custom forms from PS still aren't converted—they're building scripts for each of those. I think that's been more of a project than they anticipated.

Bringing over the old billing ended up being harder than they anticipated, as well as the historical billing. Yeah, some unanticipated issues with bringing over medications probably. That eventually got sorted out, but a lot of time on my end was spent double-checking and making sure that they were correct.

Yeah, there's always a risk of losing data. I'm always more worried about inaccurate data than missing data, actually. So we were really kind of attuned to that. We're realizing too, like other groups in our community, right? There's a doctor who used to be on our team who went to the Medical Center and went to Accuro. He didn't get any of his custom forms in that transition—they just didn't come, and there's no sort of way to get them.

Yeah, there's always a risk of losing data. I'm always more worried about inaccurate data than missing data, actually. So we were really kind of attuned to that. We're realizing too, like other groups in our community, right? There's a doctor who used to be on our team who went to the Medical Center and went to Accuro. He didn't get any of his custom forms in that transition—they just didn't come, and there's no sort of way to get them.

Some of the things would have been more ready and more smooth had we been able to wait a little bit longer. I would have preferred us to wait longer. Like, they [MYLE] made us wait as long as we were willing to risk, but that external factor put a hard stop on how long we could be.

Code	Subcodes (if applicable)	Definition
	Training	Re-training to use a new system

Transition Planning	Preparation	Work required in advance of the EMR migration
	Prioritization	Need to prioritize the EMR migration within the clinic

Examples
in person and virtually and recorded. And there’s been many, many emails sent out by them with, like, how-to steps and best practices for workflows that they identified as kind of tripping people up. Yes, there’s been a lot of support.
That’s really hard to do. I don’t think that you ever fully do it because, I mean, it’s change management, and it involves the buy-in of other people that you can’t influence fully. We had half-day in-person training sessions that were offered twice—so two half days for MOAs and two half days for physicians and clinicians—that were run by MYLE the week prior to go-live.
However, even having that, there are people who didn’t show up to any of the training, who didn’t do any of the training, who didn’t do the exercises before we went live, who obviously struggled more. But I’m not sure what more you can really do. You can’t sort of force people to do the training.
Well, I mean, we are all small business owners—we pay our own staff. So there is, of course, the cost of staff training, right? The cost of the time of staff training and the sort of loss of efficiency as staff are using a new system. You’re
I mean, MYLE has been here already. They’ve already done two more on-site visits. So one of their—like they were here for two weeks with many staff members. I guess they can tell you exactly how many. And then one person has come back for two two-day trips in order to answer people’s questions.
Formal training—we’ve had three, I think, lunchtime learning sessions with MYLE that were done both in person and virtually and recorded. And there’s been many, many emails sent out by them with, like, how-to steps and best practices for workflows that they identified as kind of tripping people up. Yes, there’s been a lot of support.
PFHT paid for them to have about the same training half days that we did, but several of them were sick. Or just didn't show up. So there's been a gap in their training for that reason , but they did have paid

responsibility to find all of the options, sort of get demos on them all, and scope them out to create comparison spreadsheets and figure out the top ones that we wanted to consider. I did that alongside a couple of other people in the group who volunteered to be on some of the demos, but I think I was the only one that did all of them. Yeah, that was before.

We were meeting, like, probably for about six months. We were meeting once a month, and then as we got to a couple of months before, we started meeting once a week. I think we were meeting every Thursday afternoon for most of the afternoon, and then as we got closer, those meetings increased as needed. Yeah, it was pretty regular.

we considered Juno, which is an Oscar-based platform out of BC. We decided not to pursue that because they didn't have, I believe, very good preventative care tracking.

know if I'm remembering this. I think they might have even come to Peterborough and done an in-person demo. I'm remembering it as—it was a while ago, though. We actually signed this contract in 2022. It's been a long time coming.
And that's because they were in development still for some Ontario features. And they really wanted to wait as long as possible before we switched so that they would be as far along the Ontario MD certification process as possible when we did the switch.

Well, yeah, they were very hard to communicate with. We actually spent probably several months before even looking at new EMRs trying to get a straight answer from TELUS about how much their ASP platform was going to cost, when we were going to be forced to make the switch, and details around what that would look like. It was very, very difficult to get.

So [Lead Administrator] is great, but she definitely wasn't picking up on the same things that I was in terms of data conversion because that's just not her job. So I think it is important to have a physician at that table if possible. It's much easier to transition to a system if the data is there properly and it's easy to

But the thing is, it was in two partitions, and your training could have been—it could be the beginning of September, not again till the end of November—like, it was spaced out. But in that time, you had time to go back and play with whatever they taught you.

You barely get a lunch, so. Right? It's long hours. It would have been nice to have training even a month in advance, not the day before.

The other thing is they need to be more organized as to how they put stuff on. Like, patient history, yes, but medication needs to be right up there at the top when it comes to organizing how things go on to the system. Because I know my boss was having—he was furious that the medications hadn't been put on before some of the other things.

Code	Subcodes (if applicable)	Definition
	Financial Cost	Financial cost of completing the EMR migration

Examples
Probably if we had stayed and moved to TELUS Cloud, we were looking at an additional $10,000 a year per doc compared to what we had been paying. So we were looking to get that cost down.
. We didn’t do that because TELUS takes about nine months right now to do that, and they charge about
So, for the weeks leading up, at least, it was probably a day and a half a week. And then the week leading up—and of course, this is all uncompensated time, right?
Probably if we had stayed and moved to TELUS Cloud, we were looking at an additional $10,000 a year per doc compared to what we had been paying. So we were looking to get that cost down.
, we didn’t even really do a demo with Accuro because their cost is basically on par with TELUS.
. So that’s kind of how we got down to MYLE. They were by far the winner in those categories, especially
I do believe that one office had to purchase a few new printers because they had the old ones. But you don’t need any really specific or expensive kind of printer to use, so that was probably like $100 of expenditure. And, like, I bought some second screens—monitors—because MYLE works really well with the
Just highlighting that the success of this project relies on physicians being willing to do unpaid work. Right? So, not mostly me, but not just me. Other people went to the demos, and we had meetings about it, and we
already alluded to his staff coming in earlier to do that, so that would—I would say it's a direct financial
billings have definitely aren’t as efficient as they were in PS as well. I know for sure I'm missing like the Q040’s and like other things that I had it, like kind of like figured out in PS. And I I don't have it—
See, I never did my own billing before. Like, I would just write it—I just had it in my schedule, and then my secretary. So, now, I'm doing it after every patient. So, that's just one other thing that I have to get my head around, to do it. So, helps my staff because they're not having to do it at the end of the day, and so it's done.
theoretical savings of $60,000 each, which is not irrelevant, right? So that then, that's it per year ongoing
I’m going to go blind because I use a laptop. It's just so small. Well, I have iMacs in my exam rooms, but at home, it’s just—I was looking at, like, this 32-inch curved screen, you know, like, what it costs. Because
They wanted to charge us—it was about $40,000—to extract data that already belonged to us to get to MYLE, and even then we would have to do the mapping process with MYLE.
Functionality and cost were really, really important. Yeah, cost was huge. I don’t think people realize—the general public doesn’t realize—that physicians are running businesses and that they are not funded by the ministry. They pay for everything: their salaries, absolutely everything for staff, leases. So, they’re running a
The EMR costs—when TELUS advised us that they were moving everybody from a solo server to an ASP platform—went up 100%. So, if we paid $40,000 a year before for EMR, we were paying $80,000 the next
Finally, I had to get angry. I got the cost, and everybody just went, “What?! How can they increase their fees
And they started charging for administrative use. They were charging for nurses. We’d never been charged for nurses before. So, there’s a $150.00 monthly license for nurses. You know, that adds up over a year as
Everything that MYLE has within the EMR is included in the price. For example, eFax. We have limitless incoming and outgoing faxes. With PSS, you have eFax, and it’s $300.00 per month.
It just became too expensive. I mean, the bottom line was TELUS has priced themselves out.
Whereas with PSS, a straight migration of one physician’s data is $1,500 plus taxes. If you want your custom forms—which are absolutely necessary—it’s another $500.00 for your custom forms.

And you don’t get billing. You get an export, and that’s extra if you want your billing. So, it can be up to $3,000 for one physician to get the data they already own.

Code	Subcodes (if applicable)	Definition
Staff Impacts	Uncertainty	Feeling of uncertainty or juggling concerns around ‘not knowing’
	Reactions	Emotional reactions or responses to the pending change, transition, etc.

Examples
But I—I think just in the process of somebody—the people who decided to use this system for us—I don't think they consulted enough people in that decision. And maybe if we were able to, like, get these different perspectives on and, like, have—or feel at least like they have a little bit of a say in what the better EMR is to go to<br><br>Yeah, I would say anxiety from staff. Yeah, absolutely. That was the biggest challenge.<br><br>So, I think the biggest shock was how little people fully understand what happens when HRM is shut off and how to manage it and ensure that everybody's getting their reports.<br>I think that was actually the scariest thing.<br>We just had a week of almost pure panic of, "Oh my god, are we getting our reports?" Nobody really seems to understand how this fully works. How can we as, like, a Family Health Organization deal with this when nobody else seems to understand it fully?
myself, I'm already more efficient in the new EMR than I was in the old one. And that's a pretty good learning curve, I think.<br><br>I think just managing the staff expectations of change. I think it's a lot harder psychologically when you're a staff member who didn't get to choose this change, right? And it's a lot of work, and now you don't know how to do anything, and you're slower, and it's more stressful. I think managing sort of the emotions of the staff was actually the hardest part.<br><br>I think some of the staff are struggling more, especially people who had maybe been using Practice Solutions since 2008 and that was the first EMR they had ever used, right? Because the workflow is different. You have to think about it differently. It doesn't work well to just try to do the same thing that you did before but in a different system. So the adaptation to that is variable.<br><br>That I didn't anticipate. Well, I knew we were going to have the support, but it actually made my staff a lot less productive when they're sitting [unclear audio] for the MYLE support people because you're going through all the MYLE stuff. Then I think I don't really anticipate that.<br><br>My nurse is looking forward to retirement. Shocked that my receptionist didn't look for a different job, so hopefully there's not a lot of [unclear speech] but that was all. She was going to retire anyways.<br><br>Anger. We all have a new line that goes, "We're on a new system, if you can just hold on for a sec while I figure this out."<br><br>. But you're—it's leading to all of this, like, your employee satisfaction just immediately plummets. I feel like everybody's home-life, work-life balance plummeted.<br><br>You know what I always felt though? I just felt like we were always dismissed, like, "We have to do this. We have to do this."—which, we do.

Code	Subcodes (if applicable)	Definition
	Vendors	Engagement with EMR vendor (old and new)

Engagement & Communication	Community Partners	Communication with community partners such as external stakeholders
	Staff	Communication with staff regarding EMR transition

Examples
And I think the major factor in that was the vendor’s motivation to do a good job and to do it with us. Like, they really invested a lot, I feel, in the process that I don’t think you would normally see.<br><br>There’s not a lot of time that the physician would normally invest in that. But MYLE was motivated to convert the data properly. It’s going to help them if they can do a good data conversion.<br><br>we also… MYLE had provided us with videos and exercises—specific exercises to try to learn the EMR before we went live—which I think was really helpful. I’ve<br><br>Yeah. So they were very, very responsive. I couldn’t have asked for a better company to work with in terms of that. It was a pretty free-flow of information. Not sure exactly what you’re looking for, but...<br><br>We were meeting, like, probably for about six months. We were meeting once a month, and then as we got to a couple of months before, we started meeting once a week. I think we were meeting every Thursday afternoon for most of the afternoon, and then as we got closer, those meetings increased as needed. Yeah, it was pretty regular.<br><br>know if I’m remembering this. I think they might have even come to Peterborough and done an in-person demo. I’m remembering it as—it was a while ago, though. We actually signed this contract in 2022. It’s been a long time coming.<br>And that’s because they were in development still for some Ontario features. And they really wanted to wait as long as possible before we switched so that they would be as far along the Ontario MD certification process as possible when we did the switch.<br><br>Until they are Ontario MD certified, they won’t integrate with HRM or OLIS—or I guess just those two. That was sort of a concern for people, but I would say it was less of a barrier than Ontario MD would want you to think.

what we’re supposed to get from HRM or by fax and sort of auditing that. Yeah, I don’t feel like there was a lot of support for that. There was sort of this semblance of trying to help, but there were a lot of balls dropped as I see it.

Well, I guess we have to get things by fax now. So that impacts the hospital in some way in their system. They can’t send it through HRM. I think that’s really it. Our system has never been connected, so yeah.

I think most organizations aren’t going to have a role in that. But I thought that Ontario MD was not that helpful. I felt like they set up HRM—they should have a way to easily turn it off. It should not be our responsibility to contact the 400 and however many people were on that spreadsheet to turn it off.

The consenting process around joining HRM should have included a description of what happens if you ever leave. I feel like we were just told, “This is great, everybody sign up, it’s so easy,” but nobody ever said, “Hey, when you leave, this is going to be a major, major issue, and we’re not going to help you with it.”

then, you know, we had a lot of difficulty communicating with PRHC in terms of trying to figure out what we’re supposed to get from HRM or by fax and sort of auditing that. Yeah, I don’t feel like there was a lot of support for that. There was sort of this semblance of trying to help, but there were a lot of balls dropped as I see it.

I think the pharmacies were working overtime.

Yeah, I mean. I’ve talked with [interviewer] already, but the two main things there I think were I don't feel very supported by Ontario MD in turning HRM off. They sent us a spreadsheet with over 400 places for us to contact individually, and I feel like they could easily have an e-mail list that they just send a message out. Like, I didn’t feel very supported by Ontario MD in terms of turning HRM off

Solutions since 2008 and that was the first EMR they had ever used, right? Because the workflow is different. You have to think about it differently. It doesn’t work well to just try to do the same thing that you did before but in a different system. So the adaptation to that is variable.

communicate between the team, make sure that everyone knew the timelines, what to expect, help people do the training, kind of whatever was needed.

However, even having that, there are people who didn’t show up to any of the training, who didn’t do any of the training, who didn’t do the exercises before we went live, who obviously struggled more. But I’m not sure what more you can really do. You can’t sort of force people to do the training.

Yeah, I mean, we first discussed it through FHO meetings after getting emails from TELUS saying that we were going to be forced onto their cloud. That’s when we started to pick it up. And I wouldn’t say that we fully decided we were going to switch before we started looking into things. We just wanted to fully assess our options before we made the commitment to go to TELUS’s ASP platform.

Code	Subcodes (if applicable)	Definition
Patient Care		Impacts of the change on patient care

Examples
have it. And OLIS is really only useful when you're looking for labs that were not copied to you or were not ordered by you, so it's not like an everyday or every-patient sort of scenario.
now it's getting better- but had to do more stuff outside of the appointment instead of during because I couldn't find it quickly during the appointment. So, I didn't want to like, have the patient just sitting there watching me do that if that makes sense. So that has probably made like the biggest - for my flow that way,
I felt like I was staring at a screen and thinking more about the EMR than I was the actual patient encounter. Because again, you're thinking where do I find this? Where am I going to go for that? How do I do this? As
Probably around 20% lighter for a week and a half.
But then the transition of how, like, the lack of training and everything, made it take longer for us to see everybody. And then if you're seeing fewer patients in a day, there's this backlog happening.
every visit is going to take longer, but the number of patients isn't going away. So if you increased—like, hired temp staff for a few weeks—sure, that's like a potential solution for when people are transitioned to a
A lot of them, I've gotten feedback of, "Oh, I can't send you messages anymore," and such. So it's just like them not being able to respond to us or just send us something, 'cause I don't know—I didn't actually use the HealthMyself—but they said a lot of them were just like, "It was nice when I could send you a message
I noticed in our practice that appointments are delayed. Yeah. And we're booking, like, 5 weeks out where we would have been 2 weeks out. Because of the different—
And if there is a problem with—again, whether this was just this migration or this system—but I think there's a risk for lab results not going through. So I had some things that needed to be very timely dealt with, and I didn't get those results. So that directly—nobody died, but they could have. Like, I'm sorry, that's
For me, because I was so busy, like, just trying to figure things out. I'd give someone an appointment and they'd come, and I'd be like, "Right, right. Where did that go?" Yeah, because you're trying to figure out how

Code	Subcodes (if applicable)	Definition
	Progress	Examples of changes or adaptations that are positive for the team/end user

Examples
We were able to choose something that we felt was built for the flow of family medicine. Like MYLE is very—it's built very differently from other EMRs. It's very inbox-focused, so getting your inbox cleared is much, much faster, and they've built it that way.<br><br>So there is a potential efficiency gain, which is one of the reasons why we chose it. But other EMRs actually… we know from the data that there's an efficiency loss even when you're at your peak performance, right? So Epic advertises that you'll be 80% as efficient as you were before. So that's not
And so, we wanted to pick something that, instead of being a legacy software that someone was going to force you to use on their server in the cloud, was kind of built to be that way. Something that could be
We were able to choose something that we felt was built for the flow of family medicine. Like MYLE is very—it's built very differently from other EMRs. It's very inbox-focused, so getting your inbox cleared is
So there is a potential efficiency gain, which is one of the reasons why we chose it. But other EMRs actually… we know from the data that there's an efficiency loss even when you're at your peak performance, right? So Epic advertises that you'll be 80% as efficient as you were before. So that's not like
I think it's true that it's like more user friendly and overall, better system, that's been helpful. That's
 Yeah, in some ways, I think certain things are faster right now. Writing notes is certainly a lot faster, I would say. Some of the things we do less often are still slow and cumbersome. It's easy, I think, it's easy to focus on the things that are harder and, you know, overlook things that are easier, but I think it's—I think there are
Just back to the last question, we don't have to run a server, which is a large expense. And, you know, if the power is off here, the EMR doesn't work anywhere. Nobody can get any work done until somebody comes down and resets the server. So, you know, there are a few new screens versus not having to run your own
I think within the last couple of weeks, I've definitely felt more confident with it. Like, getting insane—trying to find something in that moment, like patients saying, "I need to know where my Pap was last year," and I can't find it in this one. And then I would have to wait to go into PS Suite. Like, that sort of thing drives me
So, the physicians are kind of liking the faxing because they're not getting all these excessive reports that were unnecessary. And in MYLE, we have the ability to just delete, you know, fax cover pages, everything. Or you can send back if something comes in that's not yours. You can just return it and annotate it. So, I
You know, when I did contact some of the individual entities, there were a couple of radiology departments that—they would call back and tell me what I needed to send them, and they seemed to fully get it. There was no problem with them understanding what they had to do.<br>But what we didn't realize was what they didn't send.<br>Because HRM—everybody just dumps everything into HRM, and HRM just sends it. Because it's there, they have a legal liability, I suppose, to send everything, even though it's not necessarily relevant.<br>With faxing, we found out they don't send everything. But we weren't aware that they didn't send everything. They pick and choose because, obviously, it's everybody's time. And because it costs more to

Yeah, so out of all of that, certainly we found out that HRM is not the big loss we thought it was going to be because we're not getting all of the excess that you don't need.<br>So, their time in looking through their inbox is significantly reduced because they're not getting all the interim little reports that they don't need.<br>But if they're there, you have to read them. You know? You just—it's a risk if you don't because what if you miss something?<br>So now, they don't have as much pressure on them for that.<br>The functionality in MYLE—the workflow—and they're catching on to it, and they seem to really like it now.<br>But at the beginning, the workflow was a real challenge because it's a web-based EMR too, so it's a little bit
It's very different. You call, you talk to a person, and you email, and you get a response.<br>So, so far, I'm pleased with the service they provide.

Code	Subcodes (if applicable)	Definition
Lack of Progress		Examples of changes or adaptations that are negative for the team/end user

Examples
We initially tried to work with TELUS to have our data extracted, and it didn't go well.
We couldn't find those for a long time; it took about a month for them to locate those.
Things weren't coming in, so it wasn't really just a simple cutover.
We were not getting reports consistently either way
Because we really are backlogged still.
I noticed in our practice that appointments are delayed
Yeah, this—my issue apparently is unique and hasn't been figured out yet, so I can't say what it is.
But it was all changing. Like, all the data was changing all the time, so they couldn't do anything
stressful
knew that TELUS was not going to help us leave easily, and that's fine. We fully expected that. The other issue was we were left with a server that was no longer secure because TELUS notified us months before that there would be no security updates for Windows Server 12. So, we knew we had a time-limited

The other thing that was a contributing factor was there were no new, great improvements in PS. I mean, it's been the same for years. So, it's not like there was this wonderful new functionality in PSS that we were

Code	Subcodes (if applicable)	Definition
Risk Tolerance and Mitigation		Risk mitigation and management during the EMR migraiton

Examples
I think just investing the time in actually looking at the data—like the fact that I was at all the meetings and didn't just send [Lead Administrator]—is kind of what we did to mitigate the risk.
data, actually. So we were really kind of attuned to that. We're realizing too, like other groups in our community, right?
risk, but that external factor put a hard stop on how long we could be
during the transfer, largely from the hospital here, and that was tricky. So, we still, I guess you'd still go back into like the previous system, PS, and then try and find stuff that was there.
our EMR was very much locked down. We've always been really concerned about security, but it was really important that we get the data off and into a safe place to not have to worry about security breaches
because we knew we had alternatives—that we could go through ConnectingOntario to access labs and that, for HRM, we could move to faxing
impossible task. If you did not have that email list, there's no way I could sit down and contact 400 or 500 hospitals and sending facilities to ensure that—we don't have that kind of manpower
In terms of—because we're a pilot project—we knew it was never going to be perfect, but we were willing to take that risk.
we weren't getting our reports. And you worry about liability
